# Dynamics of the optimality control of transmission of infectious disease: a sensitivity analysis

**DOI:** 10.1038/s41598-024-51540-7

**Published:** 2024-01-10

**Authors:** Yasir Nadeem Anjam, Iqra Shahid, Homan Emadifar, Salman Arif Cheema, Mati ur Rahman

**Affiliations:** 1 Department of Applied Sciences, National Textile University, Faisalabad, 37610 Pakistan; 2 Department of Mathematics, Saveetha School of Engineering, Saveetha Institute of Medical and Technical Sciences, Chennai, Tamil Nadu 602105 India; 3 Department of Mathematics, Hamedan Branch, Islamic Azad University, Hamedan, Iran; 4 MEU Research Unit, Middle East University, Amman, Jordan; 5 School of Mathematical Sciences, Jiangsu University, Zhenjiang, 212013 Jiangsu People’s Republic of China; 6 Department of computer science and mathematics, Lebanese American University, Beirut, Lebanon

**Keywords:** Applied mathematics, Computational science, Scientific data, Mathematics and computing

## Abstract

Over the course of history global population has witnessed deterioration of unprecedented scale caused by infectious transmission. The necessity to mitigate the infectious flow requires the launch of a well-directed and inclusive set of efforts. Motivated by the urge for continuous improvement in existing schemes, this article aims at the encapsulation of the dynamics of the spread of infectious diseases. The objectives are served by the launch of the infectious disease model. Moreover, an optimal control strategy is introduced to ensure the incorporation of the most feasible health interventions to reduce the number of infected individuals. The outcomes of the research are facilitated by stratifying the population into five compartments that are susceptible class, acute infected class, chronic infected class, recovered class, and vaccinated class. The optimal control strategy is formulated by incorporating specific control variables namely, awareness about medication, isolation, ventilation, vaccination rates, and quarantine level. The developed model is validated by proving the pivotal delicacies such as positivity, invariant region, reproduction number, stability, and sensitivity analysis. The legitimacy of the proposed model is delineated through the detailed sensitivity analysis along with the documentation of local and global features in a comprehensive manner. The maximum sensitivity index parameters are disease transmission and people moved from acute stages into chronic stages whose value is (0.439, 1) increase in parameter by 10 percent would increase the threshold quantity by (4.39, 1). Under the condition of a stable system, we witnessed an inverse relationship between susceptible class and time. Moreover, to assist the gain of the fundamental aim of this research, we take the control variables as time-dependent and obtain the optimal control strategy to minimize infected populations and to maximize the recovered population, simultaneously. The objectives are attained by the employment of the Pontryagin maximum principle. Furthermore, the efficacy of the usual health interventions such as quarantine, face mask usage, and hand sanitation are also noticed. The effectiveness of the suggested control plan is explained by using numerical evaluation. The advantages of the new strategy are highlighted in the article.

## Introduction

The capability of infectious diseases in disrupting daily life is well documented in the existing literature of multi-disciplinary nature. In recent years, more aggressive eruptions of the viral flow are witnessed^1^. For example, the veracity of the more recent viral explosion of COVID-19 is unprecedented in modern history. To date, more than 6.8 million deaths have been reported globally due to the aforementioned virus^2^. More disturbingly, the transmission of the SARS-CoV-2 virus was accompanied by existing pathogens such as Ebola, SARS, and ZIKA. Recently, the World Health Organization (WHO) announced the onset of the newest transmissible infection called Monkeypox (MPX), named after the host of the virus^3^. More alarmingly, many researchers have noticed and documented the novel biological alterations assisting the flow of viral transmission. For example, the case of COVID-19^4^ noted consistent alterations in the gene expression of many different immune cell types. Similarly,^5^ documented the presence of EBOLA virus in epidermal and seminal vesicular tubular epithelial cells. Furthermore,^6–8^ has reported one case in Spain where an individual was detected with the co-presence of MPX, COVID-19, and HIV viruses. The scope and the coverage of research activities are mammoth while researchers from every corner of the globe are responding to the wake of infectious transmissions. A careful and tedious review of the available literature reveals that the ongoing research efforts regarding infectious diseases most commonly target three fronts such as, (i) - exploration of the clinical nature of the virus^9^, (ii) - mathematical encapsulation of the flow dynamics of the infection^10^, and (iii) - study of the socio-economic impacts of the upshots of viral flow^11^. This research is aligned with the second stream of the ongoing multi-disciplinary efforts aiming at understanding the flow dynamics of infectious transmissions.

A considerable amount of time and energy is being dedicated to understanding the mathematical nature of infectious disease transmission. For example, Biswas, launched a Susceptible Exposed Infected Recovered (SEIR) model to explore the control of infectious disease when health interventions are employed^12^. Similarly, Wangari (2015) studied the utility of the SEIR model to establish the conditions for global stability of the model when interest lies in the explanation of primary infection mechanisms^13^. Moreover, in adjacent past the consideration of optimal control strategies is gaining momentum to attain better management of the viral flow. The usage of optimal control is not restricted towards medical applications but covers numerous phenomena including, policy formulation^14^, emergency planning^15^, engineering hazard assessment^16^, and the development of more effective control program^17^. Fundamentally, an optimal control strategy is defined to assist the determining optimal protocols while dealing with complex multi-frontier problems. The resultant optimization problem is then persuaded by specifying control variables and profiling dynamics of the system under a set of given parameters, while maintaining the notion of parsimony^18^ and^19^. Many researchers have appreciated the utility of these methods to gain better control over the ongoing stochastic processes. For example, Zhilan Feng advocated the optimal control strategy in order to gain timely identification of the emergence of infectious diseases^20^. Furthermore, Kar and Soovoojeet (2016)^21^ conducted a theoretical investigation into the mathematical modeling of infectious disease with the application of optimal control^21^. Moreover, Liddo provided an elaborative account of the applicability of optimal control in demonstrating the treatment of infectious diseases^22^. Additionally^23^, Lennon-O- Naraigh and Aine Byrne studied about the Piece wise-constant optimal control strategies for controlling the outbreak of COVID-19 in the Irish population.

By using optimal control, Verma^24^ proposed and analyzed a non-linear smoking model to prevent interaction between those who are smokers and those who are smoking quitters. A mathematical model was proposed by Verma et al.^25^ to investigate the dynamics of coronavirus with lockdown effects as an epidemiological model. Moreover, the introduction of the optimal control strategy by^26^ for COVID-19, Dengue, and HIV through a mathematical model represents interactions between these infectious diseases. Further,^27^ aimed at the stability analysis of a co-circulation model for COVID-19, Dengue, and Zika, incorporating nonlinear incidence rates and vaccination strategies to enhance our understanding of the dynamic interactions among these infectious diseases. Many researchers have employed the optimal approach to model and predict the efficiency of health surveillance synergies. One may consult the account of^28^ aimed at SARS-COV-2 and HBV co-dynamics modeling, and^29^ explores the modeling of backward bifurcation and optimal control in a co-infection model for SARS-CoV-2 and ZIKV. Researchers have examined pioneer work in different areas of mathematical modeling, economics problems such as^30–33^.

Stirred by the significance of the above-documented issue, this research contributes to the existing literature on the applicability of the optimal control theory by introducing a new mathematical formation capable of explaining the dynamics of the infectious disease flow in a community. While simultaneously creating a trade between the number of infectious individuals and the cost of the considered health interventions. The whole population is classified into five mutually inclusive classes namely, susceptible class, acute infected class, chronic infected class, recovered class, and vaccinated class. Firstly, we will thoroughly examine the developed model to ensure its validity by proving pivotal delicacies such as positivity, invariant region, equilibrium points, reproduction number, and stability analysis. Along with showing complete stability analyses at equilibrium points to evaluate both local and global stability. The rationale of the optimal control scheme remains to assert plausible control law facilitating the optimization of certain performance indices. Further, the research environment is enriched by the addition of relevant control parameters such as medication, isolation, ventilation, vaccination, and quarantine levels. The existence of optimal control is proved with Pontryagin’s maximum principle (Pontryagin et al. 1962), and examine the optimal control problem to identify the required conditions for optimality. The parametric setting incorporates various scenarios.

This article is primarily alienated into six sections. Section 2 presents the qualitative analysis of the model, and Section 3 is dedicated to the sensitivity analysis of the infectious disease model, whereas, Section 4 reports the stability features of the considered model. Section 5 outlines the incorporation of optimal control with the focus of launching more feasible health interventions. Lastly, Section 6 concludes all the major findings of this study.

### Model structure

Mathematical models of infectious diseases are crucial for comprehending the patterns of disease spread and devising effective disease control strategies. Consequently, when constructing such models, emphasis should be placed on delineating the epidemiological characteristics of the disease and identifying pivotal, modifiable parameters for disease control. Numerous epidemiological models, rooted in diverse disease transmission mechanisms, have been established and documented in the literature, see^34^. These models have played a pivotal role in shaping and crafting control strategies for various diseases.

Let us consider that, for any time $t>0$, the vulnerable populations under the study, say *N*(*t*), is divided into five compartments such as Susceptible class ${\mathbb {S}}(t)$, Acute infected class $\mathbb { I}_1(t)$, Chronic infected class ${\mathbb {I}}_2(t)$, Recovered class ${\mathbb {R}}(t)$, and Vaccinated class ${\mathbb {V}}(t)$. The humans specified by ${\mathbb {S}}(t)$ are those who are at risk of contracting a specific infectious disease. Thus, the susceptible who are currently infected are moved into two classes namely Acute infected class ${\mathbb {I}}_1(t)$ and Chronic infected class ${\mathbb {I}}_2(t)$ means that if people have the time period of disease are less than six month they moved into acute infected class, if people have time period of disease beyond the six month they moved into chronic infected class, and those who are vaccinated at the rate $\sigma$ move to ${\mathbb {V}}(t)$. The vaccinated individuals may either move to Recovered class ${\mathbb {R}}(t)$ at the rate $\phi$ after receiving an infection as a result of having contact with an infectious person. Moreover, some parameters are also used in the model formulation, such as the *u* rate of vaccinated people, $\sigma$ rate of vaccinated individuals become susceptible again, $\beta$ disease transmission rate,$\gamma$ reduced the rate of the incubation period, $\rho _1$ and $\rho _2$ recovery rate from acute and chronic stages naturally, $\gamma _2$ and $\gamma _3$ recovery rate of the acute and chronic stage, *d* untreated death rate, $d_1$ disease-related death rate, $\phi$ a treated person could regain into susceptible since vaccination rate is not perfect and $\gamma _1$ people in the acute stage are moved into chronic stage.

The proposed model leverages some key assumptions grounded in biological evidence to illuminate infectious disease dynamics under optimal control strategies. One critical assumption is that the infectious disease system exhibits backward bifurcation, implying complex and non-linear interactions that contribute to sustained transmission. This assumption aligns with empirical observations in infectious disease ecology, where factors such as host heterogeneity and co-infection dynamics often lead to intricate and non-trivial system behavior. Moreover, the incorporation of optimal control strategies into the model reflects the imperative of intervention measures in disease management. Biological evidence supporting this assumption can be drawn from successful instances of interventions like vaccination campaigns and quarantine measures that have demonstrated efficacy in mitigating disease spread. While the model’s novelty or modification status is not explicitly stated, its integration of biological evidence and optimal control strategies contributes to advancing our understanding of infectious disease dynamics and intervention planning^35^.

**Figure 1:**
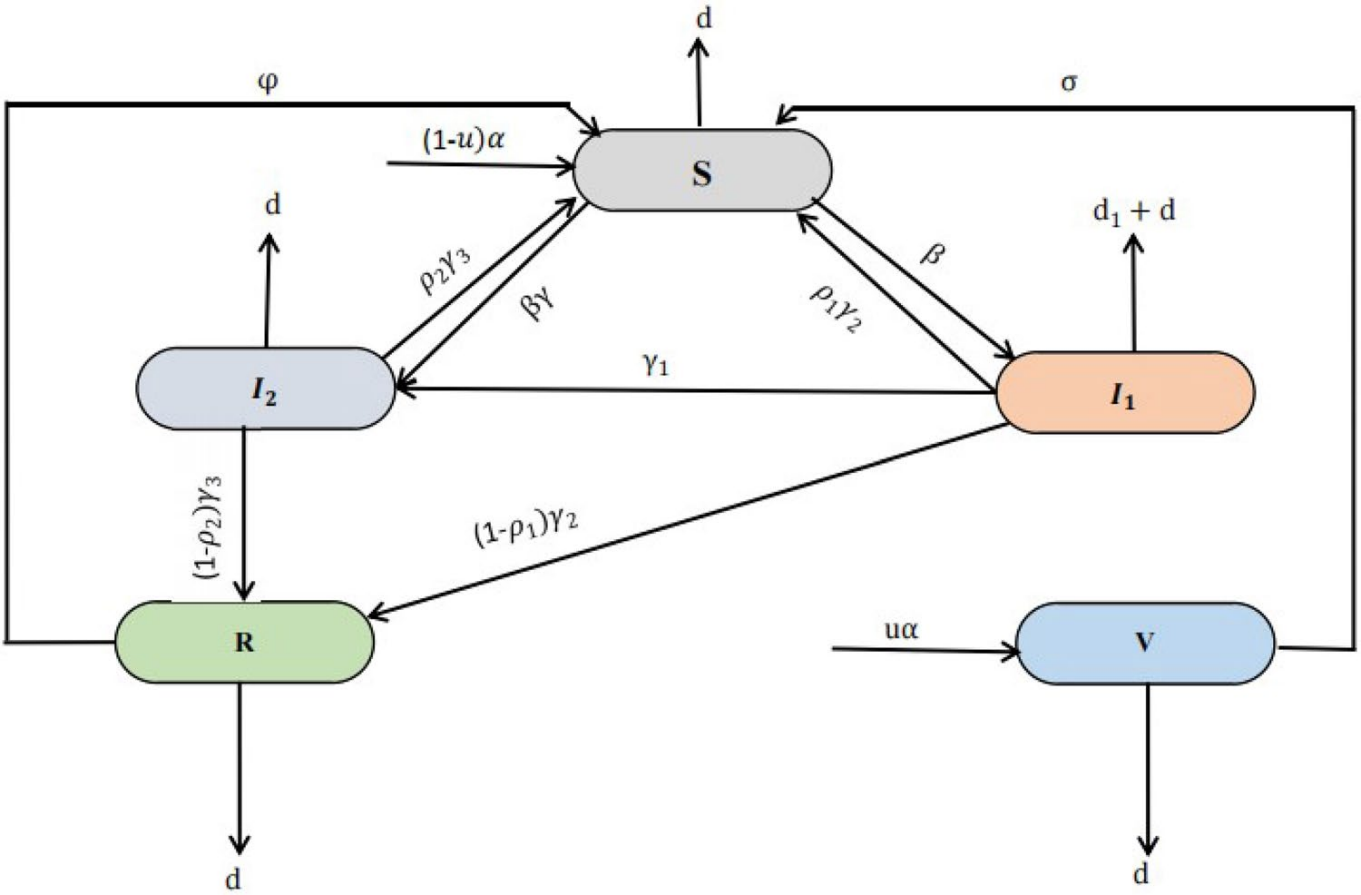
Schematic diagram illustrates the transmission dynamics of infectious disease behavior.

Therefore, based on the aforementioned considerations, the interaction flow pattern between infectious disease states is depicted in Fig. 1.

Thus, the whole human population at any time *t* is given by

$$\begin{aligned} N(t) = {\mathbb {S}}(t)+ {\mathbb {I}}_1(t)+ {\mathbb {I}}_2(t)+ {\mathbb {R}}(t)+ {\mathbb {V}}(t). \end{aligned}$$

The infectious disease model is depicted through a non-linear system of ordinary differential equations while connecting considered compartments in deterministic formation such as.

1 $$\begin{aligned} \begin{aligned}{}&\frac{d{\mathbb {S}}}{dt}=(1-u)\alpha -\beta ({\mathbb {I}}_1+\gamma {\mathbb {I}}_2){\mathbb {S}}-d{\mathbb {S}}+\phi {\mathbb {R}}+\sigma {\mathbb {V}}+\rho _1\gamma _2{\mathbb {I}}_1+\rho _2\gamma _3{\mathbb {I}}_2,\\&\frac{d{\mathbb {I}}_1}{dt}=\beta ({\mathbb {I}}_1+\gamma {\mathbb {I}}_2){\mathbb {S}}-(\gamma _1+\gamma _2+d){\mathbb {I}}_1,\\&\frac{d{\mathbb {I}}_2}{dt}=\gamma _1{\mathbb {I}}_1-(d+d_1+\gamma _3){\mathbb {I}}_2,\\&\frac{d{\mathbb {R}}}{dt}=(1-\rho _1) \gamma _2 {\mathbb {I}}_1+(1-\rho _2) \gamma _3 {\mathbb {I}}_2-(\phi +d) {\mathbb {R}},\\&\frac{d{\mathbb {V}}}{dt}=u\alpha -(\sigma +d) {\mathbb {V}}.\\ \end{aligned} \end{aligned}$$

The initial conditions are defined as follows,

$$\begin{aligned} {\mathbb {S}}(0)={\mathbb {S}}_0\ge 0,\,\, {\mathbb {I}} _1(0)={\mathbb {I}}_{1,0}\ge 0, \,\,{\mathbb {R}}(0)={\mathbb {R}}_0\ge 0,{\mathbb {I}} _2(0)={\mathbb {I}}_{2,0}\ge 0, \,\,{\mathbb {V}}(0)={\mathbb {V}}_0\ge 0.\\ \end{aligned}$$

Furthermore, the details of each term of the equations influencing the system given in the model (1) are documented in Table 1.

**Table 1:** Description of each term of the equations of the model (1).

Terms of each equations	Description
$\alpha$	Total recruitment
*u*	Rate of vaccinated people
$\sigma$	Rate of vaccinated individuals become susceptible again
$\beta$	Disease transmission rate
$\gamma$	Reduced the rate of the incubation period
$\rho _1\ \,\, \text {and}\ \,\, \rho _2$	Natural recovery rate of acute and chronic infected people
$\gamma _2\ \,\, \text {and}\ \,\, \gamma _3$	Recovery rate of acute and chronic infected people
*d*	Untreated death rate
$d_1$	Disease related death rate
$\phi$	A treated person of recovered people
$\gamma _1$	People in the acute stage are moved into chronic stage

## Qualitative analysis of the model

Here, in this section, some significant results of the proposed model, namely positivity, invariant region, equilibrium point, and basic reproduction number are given.

### Positivity

Positivity is enforced upon the initial conditions or parameters inherent in the model. It ensures that the values of the initial conditions and the parameters in the proposed model are endured greater than zero or non-negative. Therefore, reorganize the model (1) in terms is explained by

2 $$\begin{aligned} \dot{\eta }(t) = G(\eta (t)), \end{aligned}$$

where$$\,\,\,\eta (t)=(\eta _1,\eta _2,\eta _3,\eta _4,\eta _5)^T:=\big ({\mathbb {S}},{\mathbb {I}}_1, {\mathbb {I}}_2,{\mathbb {R}},{\mathbb {V}})^T, \eta (0)=({\mathbb {S}}(0),{\mathbb {I}}_1(0),\mathbb { I}_2(0),{\mathbb {R}}(0),{\mathbb {V}}(0)\big )^T\epsilon {R}^5_+$$, and

3 $$\begin{aligned} {G}(\eta ) = \begin{pmatrix} {G}_1(\eta )\\ {G}_2(\eta )\\ {G}_3(\eta )\\ {G}_4(\eta )\\ {G}_5(\eta ) \end{pmatrix} = \begin{pmatrix} (1-u)\alpha -\beta ({\mathbb {I}}_1+\gamma {\mathbb {I}}_2){\mathbb {S}}-d{\mathbb {S}}+\phi {\mathbb {R}}+\sigma {\mathbb {V}}+\rho _1\gamma _2{\mathbb {I}}_1+\rho _2\gamma _3{\mathbb {I}}_2\\ \beta ({\mathbb {I}}_1+\gamma {\mathbb {I}}_2){\mathbb {S}}-(\gamma _1+\gamma _2+d){\mathbb {I}}_1\\ \gamma _1{\mathbb {I}}_1-(d+d_1+\gamma _3){\mathbb {I}}_2\\ (1-\rho _1) \gamma _2 {\mathbb {I}}_1+(1-\rho _2) \gamma _3 {\mathbb {I}}_2-(\phi +d) {\mathbb {R}}\\ u\alpha -(\sigma +d) {\mathbb {V}} \end{pmatrix}. \end{aligned}$$

The situation is stress-free ${G}_i(\eta )\vert _{\eta _i=0}\ge 0 ,i=1,...,5$. According to the well-known Nagumo^36^ finding, the model (1) solution with an starting point $\eta _0\in R_+^{5}$, such as $\,\ \eta (t)=\eta (t;\eta _0),$ is such that $\eta (t)\in R_+^{5},\,\,\ \forall \,\ t>0$.

### Invariant region

We determined the invariant region where the solution of the proposed model is bounded. Now, consider the total population of the given model as

4 $$\begin{aligned} N(t) = {\mathbb {S}}(t) + {\mathbb {I}}_1(t) + {\mathbb {I}}_2(t) +{\mathbb {R}}(t) +{\mathbb {V}}(t). \end{aligned}$$

Differentiate (4) respecting the solution of the model (1) provides

5 $$\begin{aligned} \frac{dN}{dt} = \frac{d{\mathbb {S}}}{dt} + \frac{d\mathbb { I}_1}{dt}+\frac{d{\mathbb {I}}_2}{dt} +\frac{d{\mathbb {R}}}{dt}+ \frac{d{\mathbb {V}}}{dt}. \end{aligned}$$

Therefore, by substituting all the state equations from the model (1) in Eq. (5), then one gets

6 $$\begin{aligned} \frac{dN}{dt} = \alpha - dN. \end{aligned}$$

Taking the integration on both side of (6) with $t \rightarrow \infty$, one obtain

$$\begin{aligned} \Omega = \biggl \{{\mathbb {S}},{\mathbb {I}}_1, \mathbb { I}_2,{\mathbb {R}},{\mathbb {V}} \epsilon R_{+}^{5}: {\mathbb {S}}+ \mathbb { I}_1+{\mathbb {I}}_2+{\mathbb {R}}+{\mathbb {V}} +\le \frac{\alpha }{d}\biggl \}. \end{aligned}$$

Clearly, $\Omega$ is positively invariant, inside which the model is considered to be epidemiological meaningful and mathematically well-posed.

### Equilibrium points

Equilibrium points indicate a stable condition in the progression of the disease, where the occurrence of new infections is offset by the combined occurrences of recoveries and deaths. This section outlines the calculation of the disease-free equilibrium point and the endemic equilibrium point of the proposed system. The equilibrium points associated with the model (1) by solving $\frac{d{\mathbb {S}}}{dt} = \frac{d{\mathbb {I}}_1}{dt}= \frac{d{\mathbb {I}}_2}{dt} = \frac{d{\mathbb {R}}}{dt} = \frac{d{\mathbb {V}}}{dt} = 0$. Accordingly, the disease-free equilibrium point $F_0$, is attained by letting for ${\mathbb {I}}_1=0,\, {\mathbb {I}}_2=0,\, {\mathbb {R}}=0,\, {\mathbb {V}}=0$ such as,

$$\begin{aligned} F_0 =\big ({\mathbb {S}}_0,\,0,\,0,\,0,\,0\big )=\left( \frac{(1-u) \alpha }{\beta \gamma +d},\,0,\,0,\,0,\,0\right) , \end{aligned}$$

Now, the endemic equilibrium point is denoted as $F^*=\big ({\mathbb {S}}^*,\,{\mathbb {I}}_1^*,\,{\mathbb {I}}_2^*,\,{\mathbb {R}}^*,\,{\mathbb {V}}^*\big )$, where,

$$\begin{aligned} \begin{aligned}{}&{\mathbb {S}}^*=\frac{(1-u)d\xi +\phi \xi _1((1-\rho _1)\gamma _2{\mathbb {I}}_1^*+(1-\rho _2)\gamma _3{\mathbb {I}}_2^*)+\sigma u_1 \alpha \xi _1+\rho _2\gamma _3[(\phi +d)(\sigma +d)]}{\psi {\mathbb {I}}_2^*\xi (\beta {\mathbb {S}}-\eta )},\\&{\mathbb {I}}_1^*=\frac{ \beta \gamma {\mathbb {I}}_2 S}{\beta {\mathbb {S}}-(\gamma _1+\gamma _2+d)},\\&{\mathbb {I}}_2^*=\frac{\gamma _1 {\mathbb {I}}_1}{(d+d_1+\gamma _3)},\\&{\mathbb {R}}^*=\frac{(1-\rho _1)\gamma _2 {\mathbb {I}}_1+(1-\rho _2)\gamma _3 {\mathbb {I}}_2}{(\phi +d)},\\&{\mathbb {V}}^*=\frac{u \alpha }{\sigma +d}, \end{aligned} \end{aligned}$$

where $$\xi =(\sigma +d)(\phi +d)(d+d_1+\gamma _3),\quad \xi _1=(\sigma +d)(d+d_1+\gamma _3),\,\,\,\text {and}\,\,\, \eta = \gamma _1+\gamma _2+d, \psi = \rho _1\gamma _1\beta \gamma .$$

### The basic reproduction number

The basic reproduction number $R_0$ is a prevailing metric for evaluating the contagiousness or transmissibility of a communicable disease. It signifies the average number of individuals that are infected by one infectious individual in a susceptible population. Biologically, it reflects the contagiousness and transmissibility of a pathogen. If $R_0>1$, it indicates that the disease has the potential to sustain transmission within the population, leading to an outbreak. Conversely, if $R_0<1$, the infection is unlikely to establish a self-sustaining chain of transmission, and it may eventually die out in the population. Moreover, the reproduction number is estimated by using the method of Darraish and Wathmough^37^, and with the principle of the next-generation matrix, we have

$$\begin{aligned} F= \begin{pmatrix} -\beta {\mathbb {S}}&{} \beta \gamma {\mathbb {S}}\\ 0 &{}0\\ \end{pmatrix} \,\,\,\, \text {and}\,\,\,\, V= \begin{pmatrix} -(\gamma _1 + \gamma _2+d) &{}0\\ \gamma _1 &{}-(d+d_1+\gamma _3)\\ \end{pmatrix}. \end{aligned}$$

Therefore, the reproduction number $R_0$ for our proposed model (1) is the spectral radius of the next generation matrix $F V^{-1}$ and calculated as,

7 $$\begin{aligned} FV^{-1} = \begin{pmatrix} \Big (\frac{\beta {\mathbb {S}}}{\gamma _1+\gamma _2+d}- \frac{\beta {\mathbb {S}}\gamma \gamma _1}{(\gamma _1+\gamma _2+d)(d+d_1+\gamma _3)}\Big ) &{} \frac{\beta \gamma {\mathbb {S}}}{d+d_1+\gamma _3}\\ 0 &{} 0 \end{pmatrix}. \end{aligned}$$

Therefore, from (7), the reproduction number $R_0$ is calculated as,

8 $$\begin{aligned} R_0 = \frac{\beta \, {\mathbb {S}}_0\, \eta _2 + \beta \, {\mathbb {S}}_0\, \gamma \, \gamma _1}{\eta _1\, \eta _2}, \end{aligned}$$

where $\eta _1 = (\gamma _1+\gamma _2+d )$,  $\eta _2 = d + d_1 +\gamma _3$. The disease will spread in the population if the basic reproduction number $R_0 > 1$.

## Sensitivity analysis

The degree of influence of input parameters over the dynamics of the infectious disease model is an asset through the launch of sensitivity analysis. The sensitivity indices of the infectious disease model given in (1) are deduced by using the approach of Chitnis et al.^38^. We calculate the normalized forward sensitivity indices of a parameter *x* of $R_0$. Let

$$\begin{aligned} \Delta _x^{R_0} = \frac{\partial R_0}{\partial x} \frac{x}{R_0}. \end{aligned}$$

The estimated sensitivity projected in reproduction number with respect to various parameters is given as

$$\begin{aligned} {\left\{ \begin{array}{ll} \Delta _\beta ^{R_0} = \frac{\partial R_0}{\partial \beta } \frac{\beta }{R_0} = 1, \ \, \, \, \, \, \, \, \,\,\,\,\,\,\,\,\,\,\,\,\,\Delta _\gamma ^{R_0} = \frac{\partial R_0}{\partial \gamma } \frac{\gamma }{R_0} = 0.270,\\ \Delta _{\gamma _2}^{R_0} = \frac{\partial R_0}{\partial \gamma _2} \frac{\gamma _2}{R_0} = -0.5, \ \,\,\,\,\,\,\,\,\,\,\,\,\Delta _{\gamma _3}^{R_0} = \frac{\partial R_0}{\partial \gamma _3} \frac{\gamma _3}{R_0} = -0.876,\\ \Delta _d^{R_0} = \frac{\partial R_0}{\partial d} \frac{d}{R_0} = 0.185, \ \, \, \, \, \, \,\,\,\,\,\Delta _{d_1}^{R_0} = \frac{\partial R_0}{\partial d_1} \frac{d_1}{R_0} = -0.239,\\ \Delta _{\gamma _1}^{R_0} = \frac{\partial R_0}{\partial \gamma _1} \frac{\gamma _1}{R_0} = 0.439. \end{array}\right. } \end{aligned}$$

**Table 2:** Sensitivity indices and parameters of the reproduction number $R_0$.

Parameters	Sensitivity indices	Parameters	Sensitivity indices
$\beta$	+	$\gamma$	+
$\gamma _1$	+	$\gamma _2$	–
*d*	+	$\gamma _3$	–
$d_1$	–	$\gamma _1$	+

Table 2 comprehends the directional projection of the parameters involved in the considered model explaining the viral infectious disease transmission flow. The existence of a positive relationship is voted through the plus sign whereas the negative sign avows the showing of the negative relationship between parameters and the transmission rate. One may be noted that an increase in the reproduction number remains associated with an increase in parameters such as disease transmission rate, reduced incubation period, rate of transfer from the acute stage to the chronic stage, and untreated death rate. Whereas, the reproduction number is estimated to be negatively associated with parameters such as disease-related death rate, and recovery rates belonging to the acute stage and chronic stage.

**Figure 2:**
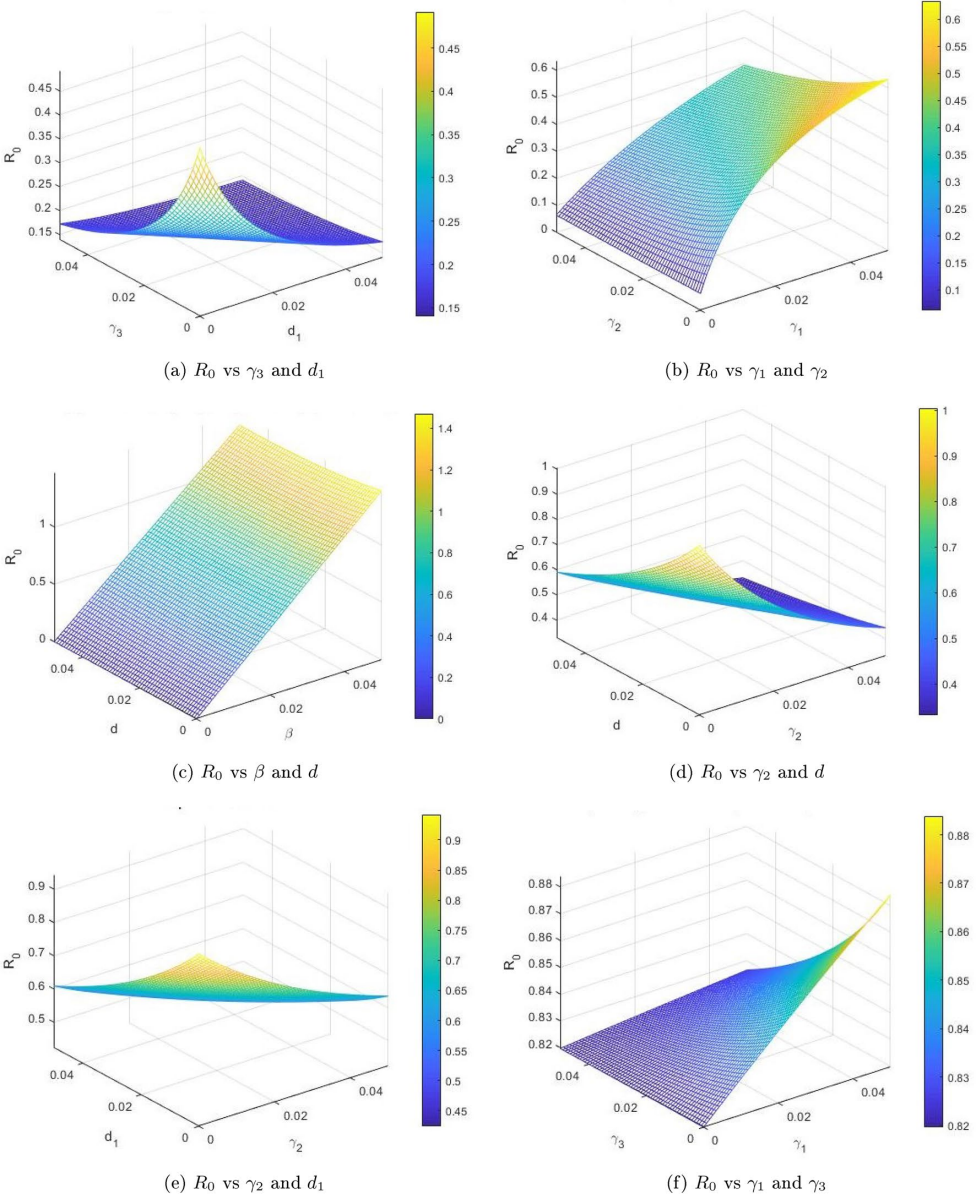
The graphical result shows the dynamics of various compartmental parameters and their effects on the reproduction number $R_0$.

Figure 2 shows the dynamical behavior of the sensitivity analysis of the basic reproduction number $R_0$, in the context of the disease transmission. Moreover, this analysis helps us to understand the significance of various factors that play a vital role in the transmission of infectious diseases. We plotted the reproduction number $R_0$ as a function of $\gamma _3, d_1, \gamma _1, \gamma _2, \beta$ and *d*. Figure 2a shows the effect of effectual disease-related death rate and recovery rate of chronic infected patients on the reproduction number, we can see that these two parameters may increase to $R_0$ greater than unity if the value of $\gamma _3$ remain up to greater than 1 and the recovery rate also remains greater than 1. The combined effect of transmission and recovery rate on $R_0$ is shown in Fig. 2b. As one parameter $\beta$ is directly proportional and $\gamma _1$ is inversely related to $R_0$, thus we can see that the basic reproduction number value may be reduced below unity even if the transmission rate $\beta$ approaches 1 and the recovery rate of acute and chronic infected increases. Figure 2c shows the effect of transmission rate $\beta$ and *d*. We can see that these two parameters have a considerable impact in reducing or increasing the value of the basic reproduction number $R_0$. Furthermore, Fig. 2d shows the effect of recovery rate $gamma_2$ and disease-related death rate *d*, and also Fig. 2e shows the inverse relationship between $R_0$ and parameters. Similarly, Fig. 2f shows the impact of reducing or increasing the value of reproduction number $R_0$.

## Stability analysis

In this section, we analyze the local and global stabilities of the proposed model at equilibrium points. Local stability is often determined by examining the eigenvalues of the Jacobian matrix evaluated at the equilibrium point. If all eigenvalues have negative real parts, the equilibrium is locally stable. Global stability considers the system’s behavior across its entire range, often requiring more advanced mathematical techniques such as Lyapunov analysis.

### Local stability analysis

The aim of conducting local stability analysis is to determine whether a minor disturbance in the disease system would lead to the persistence or elimination of the disease. In the realm of epidemiological models, comprehending the concept of local stability at equilibrium points holds significance. It provides insights into the dynamics of infectious diseases and their transmission within a population. The endemic equilibrium point and disease-free equilibrium point, which are locally asymptotically stable, are illustrated in the following theorem.

#### Theorem 4.1

The disease-free equilibrium point $F_0$ is locally asymptotically stable when $R_0$ < 1, however, when $R_0>1$, it is unstable.

#### Proof

The Jacobin matrix of disease-free equilibrium point $F_0$ for the proposed model is given as

9 $$\begin{aligned} J(F_0)= \begin{pmatrix} -\beta ({\mathbb {I}}_1+\gamma {\mathbb {I}}_2)-d &{}- \beta {\mathbb {S}}_0+\rho _1\gamma _2 &{} \beta \gamma {\mathbb {S}}_0+\rho _2 \gamma _3 &{} \phi &{}0\\ -\beta ({\mathbb {I}}_1+\gamma {\mathbb {I}}_2) &{} \beta {\mathbb {S}}_0-(\gamma _1+\gamma _2+d) &{} \beta \gamma {\mathbb {S}}_0 &{} 0&{}0 \\ 0 &{} \gamma _1 &{} -(d + d_1 +\gamma _3)&{}0&{}0\\ 0 &{} (1-\rho _1)\gamma _2 &{} (1-\rho _2)\gamma _3 &{} -(\phi +d)&{}0 \\ 0&{}0&{}0&{}0&{}-(\sigma +d) \end{pmatrix}. \end{aligned}$$

The evaluation of the jacobian at drinking-free equilibrium provides

10 $$\begin{aligned} J(F_0)= \begin{pmatrix} -d &{}- \beta {\mathbb {S}}_0+\rho _1\gamma _2 &{} \beta \gamma {\mathbb {S}}_0+\rho _2 \gamma _3 &{} \phi &{}0\\ 0 &{} \beta {\mathbb {S}}_0-(\gamma _1+\gamma _2+d) &{} \beta \gamma {\mathbb {S}}_0 &{} 0&{}0 \\ 0 &{} \gamma _1 &{} -(d + d_1 +\gamma _3)&{}0&{}0\\ 0 &{} (1-\rho _1)\gamma _2 &{} (1-\rho _2)\gamma _3 &{} -(\phi +d)&{}0 \\ 0&{}0&{}0&{}0&{}-(\sigma +d) \end{pmatrix}. \end{aligned}$$

With the aid of the Jacobin matrix of the disease-free equilibrium point, the resultant eigenvalues are $\lambda _1 = -\beta (\mathbb { I}_1+\gamma {\mathbb {I}}_2)< 0, \lambda _2 = -(\sigma +d) < 0,$ whereas,

$$\begin{aligned} \begin{vmatrix} J(F_0)-\lambda I \end{vmatrix} = \begin{vmatrix} \beta {\mathbb {S}}_0-(\gamma _1+\gamma _2+d)-\lambda&\beta \gamma {\mathbb {S}}_0&0\\ \gamma _1&-(d+d_1+\gamma _3)-\lambda&0\\ (1-\rho _1)\gamma _2&(1-rho_2)\gamma _3&-(\phi +d)-\lambda \end{vmatrix}=0, \end{aligned}$$

$\lambda _4 =-(d+d_1+\gamma _3),\, \lambda _5 = -(\phi +d), \text {and} \, \lambda _3= \beta {\mathbb {S}}_0-(\gamma _1+\gamma _2+d) <0$. $\lambda _1, \lambda _2, \lambda _4, \text {and} \lambda _5$ are real and negative. Further, $\lambda _3$ is negative and real if $R_0 < 1$. Using the Routh-Hurwitz criterion^39^, it is verified that, each eigenvalue of the polynomial equation contains a non-positive real parts when $R_0<1$. As a result, the disease-free equilibrium point $F_0$ is locally asymptotically stable. $\square$

#### Theorem 4.2

The endemic equilibrium point $F^*$ will be locally asymptotically stable if the value of $R_0$ > 1, however, when $R_0 < 1$, it is unstable.

#### Proof

The determination of the Linearization for model (1) at the endemic equilibrium point $F^*$ is carried out by

$$\begin{aligned} J(F^*)= \begin{pmatrix} -\beta ({\mathbb {I}}_1+\gamma {\mathbb {I}}_2)-d &{}- \beta {\mathbb {S}}+\rho _1\gamma _2 &{} \beta \gamma {\mathbb {S}}+\rho _2 \gamma _3 &{} \phi &{}0\\ -\beta ({\mathbb {I}}_1+\gamma {\mathbb {I}}_2) &{} \beta {\mathbb {S}}-(\gamma _1+\gamma _2+d) &{} \beta \gamma {\mathbb {S}} &{} 0&{}0 \\ 0 &{} \gamma _1 &{} -(d + d_1 +\gamma _3)&{}0&{}0\\ 0 &{} (1-\rho _1)\gamma _2 &{} (1-\rho _2)\gamma _3 &{} -(\phi +d)&{}0 \\ 0&{}0&{}0&{}0&{}-(\sigma +d) \end{pmatrix}. \end{aligned}$$

By using row matrix transformation the following matrix is given by:

11 $$\begin{aligned} J(F^*)= \begin{pmatrix} -\mu &{}- 0 &{} 0 &{} 0 &{}0\\ -\beta ({\mathbb {I}}_1+\gamma {\mathbb {I}}_2) &{} -\mu _1 &{} 0 &{} 0&{}0 \\ 0 &{} \gamma _1 &{} -\mu _2&{}0&{}0\\ 0 &{} (1-\rho _1)\gamma _2 &{} (1-\rho _2)\gamma _3 &{}-\mu _3 &{}0 \\ 0&{}0&{}0&{}0&{}-\mu _4&{} \end{pmatrix}. \end{aligned}$$

where, let $\mu =\beta ({\mathbb {I}}_1+\gamma {\mathbb {I}}_2)-d, \ \,\, \mu _1=(\gamma _1+\gamma _2+d), \ \,\, \mu _2 = (d + d_1 +\gamma _3), \ \,\, \mu _3 =(\phi +d), \ \,\,\text {and}\ \,\, \mu _4=(\sigma +d).$ It is clear that all of eigenvalues values of $J(F^*)$ have negative and real if $R_0 > 1$. $\square$

### Global stability analysis

Global stability in a disease model examines whether, over the entire range of conditions, the system converges to a stable state in terms of disease behavior. It is crucial for understanding the persistent, long-term dynamics and overall prevalence of drinking in a population. The endemic equilibrium point and disease-free equilibrium point, which are locally asymptotically stable, are illustrated in the subsequent theorem.

#### Theorem 4.3

The disease free equilibrium point $F_0$ is globally asymptotically stable for $R_0 < 1$, otherwise, unstable for $R_0 > 1$.

#### Proof

The following Lyapunov function has been properly defined and satisfies the condition of being positive definite and its derivative is negative definite. By focusing on infected and susceptible individuals, the Lyapunov function effectively quantifies the balance between disease transmission and population susceptibility, providing insights into the long-term behavior and stability of the epidemic model. To show the result, Firstly, we state the Lyapunov function is specified by,

12 $$\begin{aligned} L = f_1 {\mathbb {S}} + f_2 {\mathbb {I}}_1 + f_3 {\mathbb {I}}_2, \end{aligned}$$

Where $f_1, f_2\, and\, f_3$ are positive constant and derivative of L is given by

$$\begin{aligned} L^{'}= & {} f_1 {\mathbb {S}}^{'} +f_2 {\mathbb {I}}_1^{'} +f_3 {\mathbb {I}}_2^{'},\\ L^{'}= & {} f_1\bigg \{(1-u)\alpha -\beta ({\mathbb {I}}_1 +\gamma {\mathbb {I}}_2){\mathbb {S}}_0-d{\mathbb {S}}_0 +\phi {\mathbb {R}}+\sigma {\mathbb {V}} +\rho _1 \gamma _2 {\mathbb {I}}_1 + \rho _2\gamma _3 {\mathbb {I}}_2\bigg \}\\{} & {} +f_2\bigg \{\beta ({\mathbb {I}}_1+\gamma {\mathbb {I}}_2){\mathbb {S}}_0-(\gamma _1+\gamma _2+d)\bigg \} +f_3\bigg \{\gamma _1\mathbb { I}_1-(d+d_1+\gamma _3){\mathbb {I}}_2\bigg \}, \end{aligned}$$

Now, by arranging the above terms yield,

$$\begin{aligned} \begin{aligned} L^{'}&=\bigg \{f_1{-\beta ({\mathbb {I}}_1 +\gamma {\mathbb {I}}_2)} +f_3 \beta ({\mathbb {I}}_1+\gamma {\mathbb {I}}_2)-f_1 d\bigg \}({\mathbb {S}}-{\mathbb {S}}_0)+\bigg \{f_1\rho _1\gamma _2-f_2(\gamma _1+\gamma _2+d)\\&\quad +f_3\gamma _1\bigg \}{\mathbb {I}}_1+\bigg \{f_1\rho _2\gamma _3-(d+d_1+\gamma _3)\bigg \}{\mathbb {I}}_2+\phi {\mathbb {R}} f_1 +\sigma {\mathbb {V}} f_1. \end{aligned} \end{aligned}$$

where $F_0=\frac{(1-u) \alpha }{\beta \gamma +d}$. Further, for ${\mathbb {S}}$ > ${\mathbb {S}}^0$ and $R_0$ < 1, then $L^{'}(t)<0$. Also if ${\mathbb {S}}= {\mathbb {S}}^0$ then $L^{'}(t) = 0$. By Lasla invarience principle^40,41^, ${\mathbb {I}}_1 = {\mathbb {I}}_2 = 0.$ The Lyapunov function conditions are all positive definite, the function is positive definite and its derivative is negative definite. Therefore the disease-free equilibrium $F_0$ is globally asymptotically stable. $\square$

#### Theorem 4.4

The endemic equilibrium point $F^*$ is globally asymptotically stable, if the value of $R_0 > 1$, however, when $R_0 < 1$, it is unstable.

#### Proof

In order to prove the global stability of the proposed model (1) at the endemic equilibrium point $F^*$, the Castilo Chevez method is used from^42,43^. Further, we consider (1) as:

13 $$\begin{aligned} \begin{aligned}{}&\frac{d{\mathbb {S}}}{dt}=(1-u)\alpha -\beta ({\mathbb {I}}_1+\gamma {\mathbb {I}}_2){\mathbb {S}}-d{\mathbb {S}}+\phi {\mathbb {R}}+\sigma {\mathbb {V}}+\rho _1\gamma _2{\mathbb {I}}_1+\rho _2\gamma _3{\mathbb {I}}_2,\\&\frac{d{\mathbb {I}}_1}{dt}=\beta ({\mathbb {I}}_1+\gamma {\mathbb {I}}_2){\mathbb {S}}-(\gamma _1+\gamma _2+d){\mathbb {I}}_1,\\&\frac{d{\mathbb {I}}_2}{dt}=\gamma _1 {\mathbb {I}}_1-(d+d_1+\gamma _3){\mathbb {I}}_2. \end{aligned} \end{aligned}$$

Taking the jacobian and the additive compound matrix of order 2 for the Eq. (13), get the following matrix,

14 $$\begin{aligned} J= & {} \begin{pmatrix} -a_1{_1} &{} a_1{_2} &{} a_1{_3} \\ a_2{_1} &{} -a_2{_2} &{} 0 \\ 0 &{} a_{3_2} &{} -a_3{_3} \\ \end{pmatrix},\nonumber \\ J^{|2|}= & {} \begin{pmatrix} -(a_1{_1}+a_2{_2}) &{} a_2{_3} &{} -a_1{_3} \\ a_3{_2} &{} -(a_1{_1}+a_2{_2}) &{} a_1{_2} \\ -a_3{_1} &{} a_2{_1} &{} -(a_2{_2}+a_3{_3}) \\ \end{pmatrix}. \end{aligned}$$

Consider a function $Q(x) = Q\,({\mathbb {S}}, {\mathbb {I}}_1, {\mathbb {I}}_2) =diag\bigg \{{ \frac{{\mathbb {S}}}{{\mathbb {I}}_1},\frac{{\mathbb {S}}}{{\mathbb {I}}_1},\frac{{\mathbb {S}}}{{\mathbb {I}}_1}}\bigg \}, then \,Q^{-1}(x)= diag\bigg \{\frac{{\mathbb {I}}_1}{{\mathbb {S}}},\frac{{\mathbb {I}}_1}{{\mathbb {S}}},\frac{{\mathbb {I}}_1}{{\mathbb {S}}}\bigg \}$, then the time derivative of the function, $Q_f(x)$, suggests that

15 $$\begin{aligned} Q_f(x)=diag\left\{ \frac{\mathbb {\dot{S}}}{{\mathbb {I}}_1}-\frac{{\mathbb {S}} \mathbb {\dot{I}}_{1}}{{\mathbb {I}}_{1}^{2}},\frac{\mathbb {\dot{S}}}{{\mathbb {I}}_{1}}-\frac{{\mathbb {S}} \mathbb {\dot{I}}_{1}}{{\mathbb {I}}_{1}^{2}},\frac{\mathbb {\dot{S}}}{{\mathbb {I}}_1}-\frac{{\mathbb {S}} \mathbb {\dot{I}}_{1}}{{\mathbb {I}}_{1}^{2}}\right\} . \end{aligned}$$

Now $Q_f(x) Q^{-1} = diag \bigg \{\frac{\dot{{\mathbb {S}}}}{{\mathbb {S}}}-\frac{\dot{{\mathbb {I}}_1}}{{\mathbb {I}}_1},\frac{\dot{{\mathbb {S}}}}{{\mathbb {S}}}-\frac{\dot{{\mathbb {I}}_1}}{{\mathbb {I}}_1},\frac{\dot{{\mathbb {S}}}}{{\mathbb {S}}}-\frac{\dot{{\mathbb {I}}_1}}{{\mathbb {I}}_1}\bigg \}$ and $Q J_2^{|2|} Q^{-1} = J_2^{|2|}$. $A = Q_f Q^{-1} + Q J_2^{|2|} Q^{-1}$, which can be described as

16 $$\begin{aligned} A= \begin{pmatrix} A_{11} &{} A_{12}\\ A_{21} &{} A_2{2}\\ \end{pmatrix}, \end{aligned}$$

where

$$\begin{aligned} {\left\{ \begin{array}{ll} A_1{_1} = \frac{\dot{S}}{S} - \frac{\dot{I_1}}{I_1} - \beta (I_1 + \gamma I_2) - (\gamma _1 + \gamma _2 +d),\\ A_1{_2} = \begin{pmatrix} 0 &{} \rho _2 \gamma _3\\ \end{pmatrix},\\ A_2{_1} = \begin{pmatrix} \gamma _1\\ 0 \end{pmatrix},\\ A_2{_2} = \begin{pmatrix} x_1{_1} &{} x_1{_2}\\ x_2{_1} &{} x_2{_2} \end{pmatrix},\\ x_1{_1} = \frac{\mathbb {\dot{S}}}{{\mathbb {S}}} -\frac{\mathbb {\dot{I}}_1}{{\mathbb {I}}_1} - \beta ({\mathbb {I}}_1 + \gamma {\mathbb {I}}_2) -(\gamma _1+\gamma _2+d),\\ x_1{_2} = \rho _1\gamma _2,\\ x_2{_1} = \beta ({\mathbb {I}}_1 + \gamma {\mathbb {I}}_2),\\ x_2{_2} = \frac{\mathbb {\dot{S}}}{{\mathbb {S}}} - \frac{\mathbb {\dot{I}}_1}{{\mathbb {I}}_1} -(\gamma _1+\gamma _2+d) - (d+d_1+\gamma _3).\\ \end{array}\right. } \end{aligned}$$

Let $(b_1,b_2,b_3)$ be a vector in $R^3$ and the $||\cdot ||$ of $(b_1,b_2,b_3)$ is specified by $||b_1,b_2,b_3|| = \max \{||b_1||+||b_2||+||b_3||\}.$ Now, we yield the Lozinski measure defined by^44^, $l(A) \le \sup \{h_1,h_2\} = \sup \{l(A_1{_1})+||(A_1{_2})||, l(A_2{_2})+||(A_2{_1})||\},$ where $h_i = l(A_i{_i}) + ||(A_i{_j})||$ for $i = 1,2$ and $i \ne j,$ which implies that

17 $$\begin{aligned} h_1 = l(A_1{_1})+||(A_1{_2})||,\, h_2 = l(A_2{_2}) +||(A_2{_1})||, \end{aligned}$$

where $$l(A_1{_1}) = \frac{\mathbb {\dot{S}}}{{\mathbb {S}}} - \frac{\mathbb {\dot{I}}_1}{{\mathbb {I}}_{1}}-\beta ({\mathbb {I}}_1 +\gamma {\mathbb {I}}_2) - (\gamma _1 + \gamma _2+ d)$$,

$l(A_2{_2}) = \max \bigg \{\frac{\mathbb {\dot{S}}}{{\mathbb {S}}} - \frac{\mathbb {\dot{I}}_1}{{\mathbb {I}}_1} - \beta ({\mathbb {I}}_1 + \gamma {\mathbb {I}}_2)-(\gamma _1+\gamma _2+d), \ \,\beta (\mathbb { I}_1+\gamma {\mathbb {I}}_2)\bigg \} = \bigg \{\frac{\mathbb {\dot{S}}}{{\mathbb {S}}} -\frac{\mathbb {\dot{I}}_1}{{\mathbb {I}}_1}-\beta ({\mathbb {I}}_1+\gamma {\mathbb {I}}_2)-(\gamma _1+\gamma _2+d) \bigg \} , ||(A_1{_2})|| = \rho _2 \gamma _3$ and $||(A_2{_1})|| = \max \{\gamma _1,0\} = \gamma _1$. Therefore, $h_1$ and $h_2$ becomes, such that $h_1 \le \frac{\mathbb {\dot{S}}}{{\mathbb {S}}}-(\gamma _1+\gamma _2+d)-(d+d_1+\gamma _3)$ and $h_2 \le \frac{\mathbb {\dot{S}}}{{\mathbb {S}}} -\gamma _1- \min {\gamma _1,\gamma _2}$, which shows that $l(A) \le \bigg \{\frac{\mathbb {\dot{S}}}{{\mathbb {S}}}-\gamma _1-\min \{\gamma _1,\gamma _2\}+\gamma _2\bigg \}$. Hence, $l(A) \le \frac{\mathbb {\dot{S}}}{{\mathbb {S}}}-\gamma _1$. Taking integral of *l*(*A*), one get

18 $$\begin{aligned}{} & {} \lim _{t \rightarrow \infty } \sup \sup \frac{1}{t} \int _{0}^{\infty } l(A)dt<-\gamma _1,\nonumber \\{} & {} k^{~} = \lim _{t \rightarrow \infty } \sup \sup \frac{1}{t} \int _{0}^{t} l(A)dt<0. \end{aligned}$$

Hence the model (1) is globally asymptotically stable. $\square$

### Numerical results of stability analysis

The Runge-Kutta method is widely employed in epidemiological models for numerical simulation due to its robustness and accuracy in solving differential equations that govern disease dynamics. Epidemiological models often involve a system of coupled differential equations that describe the interactions between various population compartments, such as susceptible, infected, and recovered individuals. The Runge-Kutta fourth-order method is renowned for its high accuracy in approximating solutions of differential equations, providing reliable and precise numerical results. Additionally, RK-4 exhibits strong convergence properties, ensuring stability and efficiency in solving a wide range of mathematical problems. Its versatility makes it suitable for capturing the complex and dynamic nature of infectious disease spread, allowing researchers to explore different scenarios and intervention strategies. The RK method’s efficiency and accuracy contribute to the reliability of numerical simulations, aiding in the understanding and prediction of epidemiological dynamics essential for public health decision-making.

In this section, we used the fourth-order Runge-Kutta method to solve the deterministic mathematical model and verify the analysis^45^. Additionally, the use of numerical simulations can provide valuable insights into the dynamic behavior of the model. However, it’s important to note that the selection of parameter approximations should be justified and explained in detail. Additionally, it would be useful to discuss any potential limitations or assumptions made in using the Runge-Kutta method and how it affects the accuracy of the results. Variables and parametric values are given in Table 3, which are used for simulations. Furthermore, the time interval is taken from 0 - 200 units with initial problems for Susceptible class ${\mathbb {S}}(t)$, Acute infected class ${\mathbb {I}}_1(t)$, Chronic infected class ${\mathbb {I}}_2(t)$, Vaccinated class ${\mathbb {V}}(t)$, and Recovered class ${\mathbb {R}}(t)$. The applications of the Runge-Kutta method of fourth order on the proposed model lead to the following system:

$$\begin{aligned} {\left\{ \begin{array}{ll} \frac{{\mathbb {S}}^{i+1} - {\mathbb {S}}^i}{l} = (1-u)\alpha -\beta ({\mathbb {I}}_1^{i}+\gamma {\mathbb {I}}_2^{i}) {\mathbb {S}}^{i+1}-dS^{i+1}+\sigma {\mathbb {V}}^{i+1}+ \rho _1 \gamma _2 {\mathbb {I}}_1^{i+1} +\rho _2 \gamma _3 {\mathbb {I}}_2^{i+1},\\ \frac{{\mathbb {I}}_1^{i+1} - {\mathbb {I}}^i}{l} = \beta ({\mathbb {I}}_1^{i+1}+\gamma {\mathbb {I}}_2^{i+1}) {\mathbb {S}}^{i+1}- (\gamma _1+\gamma _2+d) {\mathbb {I}}_1^{i+1},\\ \frac{{\mathbb {I}}_2^{i+1} - {\mathbb {I}}^i}{l} = \gamma _1 {\mathbb {I}}_1^{i+1} - (d+d_2+\gamma _3) {\mathbb {I}}_2^{i+1},\\ \frac{{\mathbb {R}}^{i+1} - {\mathbb {R}}^i}{l} = (1-\rho _1)\gamma _2 {\mathbb {I}}_1^{i+1}+(1-\rho _2)\gamma _3 {\mathbb {I}}_2^{i+1} - (\sigma +d) {\mathbb {R}}^{i+1},\\ \frac{{\mathbb {V}}^{i+1} - {\mathbb {V}}^i}{l} = u\alpha -(\sigma +d){\mathbb {V}}^{i+1}.\\ \end{array}\right. } \end{aligned}$$

### Algorithm

Step 1: $${\mathbb {S}}(0)=0$$, $${\mathbb {I}}_1(0)=0$$, $${\mathbb {I}}_2(2) =0$$, $${\mathbb {R}}(0)=0$$, $${\mathbb {V}}(0)=0$$.

Step 2: for i=1,2,3,...,n-1.

$$\begin{aligned} {\left\{ \begin{array}{ll} {\mathbb {S}}^{i+1} = \frac{l\,(1-u)\,\alpha -l\,\beta \, ({\mathbb {I}}_1^{i}\,+\,\gamma \, {\mathbb {I}}_2^{i})\,+\,l\,\sigma \, {\mathbb {V}}^{i+1}}{l\beta \, ({\mathbb {I}}_1^{i}\,+\,\gamma \, {\mathbb {I}}_2^{i})\,-d},\\ {\mathbb {I}}_1^{i+1} = \frac{{\mathbb {I}}_1^{i} +l\beta {\mathbb {I}}_1^{i}\gamma {\mathbb {S}}^{i}}{1-l \,\beta (({\mathbb {S}}^{i}+l\,(\gamma _1+\gamma _2+d)},\\ {\mathbb {I}}_2^{i+1} = \frac{l\gamma _1 I_1^{i}+I_2^{i}}{1+l\,(d+d_1+\gamma _3)},\\ {\mathbb {R}}^{i+1} = \frac{l\,(1-\rho _1)\gamma _2 {\mathbb {I}}_1^{i}+l\,(1-\rho _2)\gamma _3 {\mathbb {I}}_2^{i+1}+{\mathbb {R}}^i}{1+l(\phi +d)},\\ {\mathbb {V}}^{i+1} = \frac{l u \alpha + {\mathbb {V}}^{i}}{1+l(\sigma +d)}.\\ \end{array}\right. } \end{aligned}$$

Step 3: for $i= 1, 2, 3,...,n-1$, write ${\mathbb {S}}^*(t_i)={\mathbb {S}}^*$, ${\mathbb {I}}_1^*(t_i)={\mathbb {I}}_1^*$, ${\mathbb {I}}_2^*(t_i)={\mathbb {I}}_2^*$, ${\mathbb {R}}^*(t_i)={\mathbb {R}}^*$, ${\mathbb {V}}^*(t_i)={\mathbb {R}}^*$.

**Figure 3:**
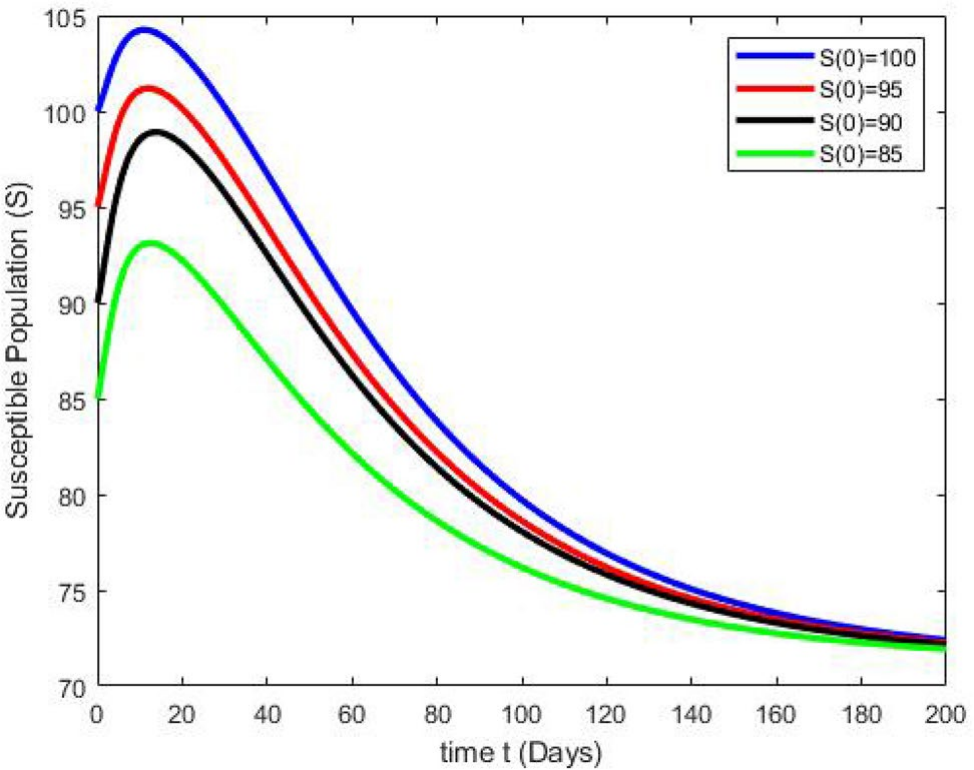
The effects of variations in the Susceptible class ${\mathbb {S}}(t)$ are described by simulation results.

**Figure 4:**
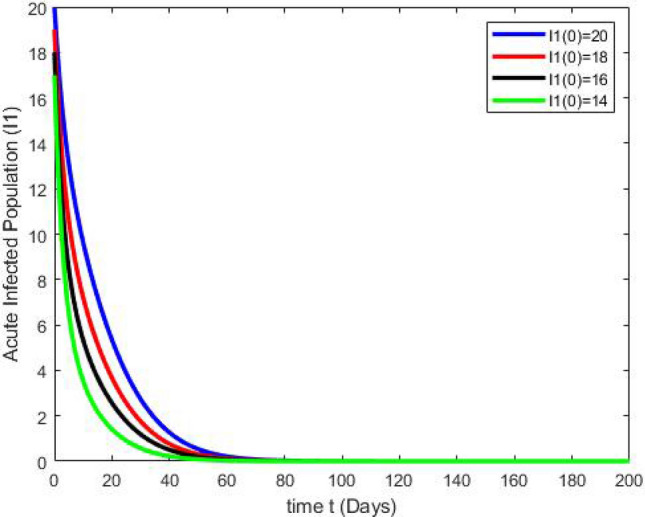
The effects of variations in the Acute Infected class ${\mathbb {I}}_1(t)$ are described by simulation results.

**Figure 5:**
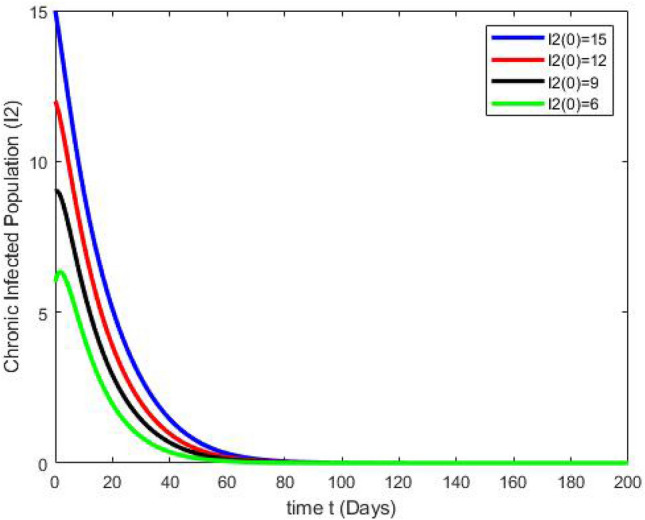
The effects of variations in the Chronic Infected class ${\mathbb {I}}_2(t)$ are described by simulation results.

**Figure 6:**
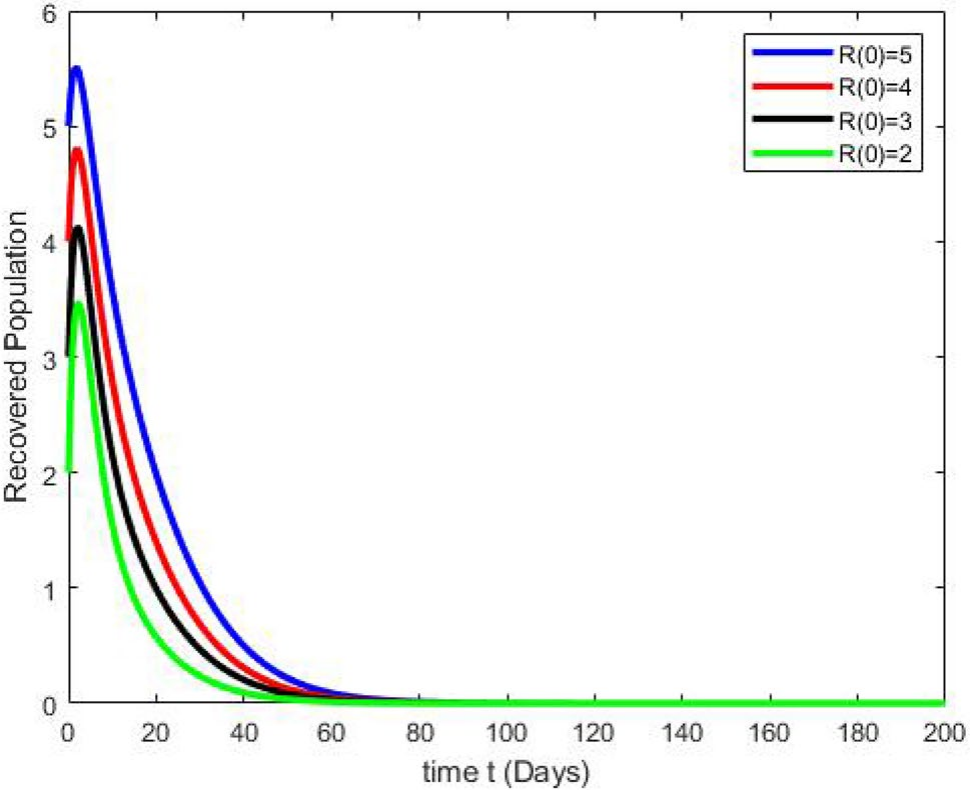
The effects of variations in the Recovered class ${\mathbb {R}}(t)$ are described by simulation results.

**Figure 7:**
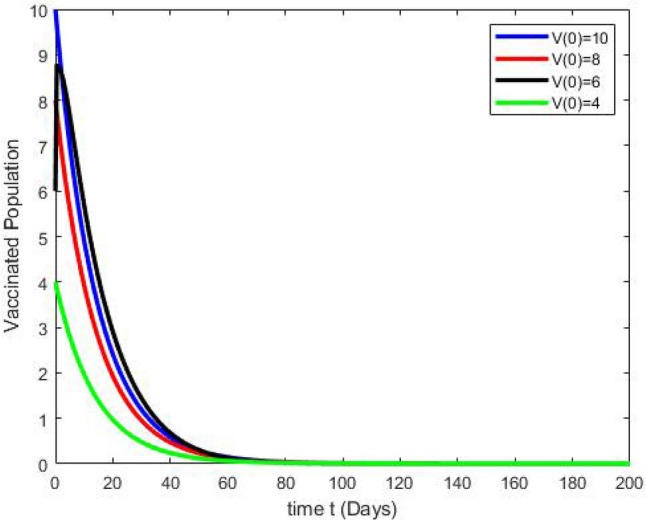
The effects of variations in the Vaccinated class ${\mathbb {V}}(t)$ are described by simulation results.

By running the above findings with the aid of the Matlab software, the graphs presented in the Figs. 3, 4, 5, 6, 7, which shows the dynamic behavior of Susceptible class ${\mathbb {S}}(t)$, Acute infected class ${\mathbb {I}}_1(t)$, Chronic infected class ${\mathbb {I}}_2(t)$, Vaccinated class ${\mathbb {V}}(t)$ and Recovered class ${\mathbb {R}}(t)$.

Figures 3, 4, 5, 6, 7 present the dynamics of the stability behavior of all considered classes of the population while taking into account all the employed initial conditions. The magnitude of the susceptible class is varied for values such as 100, 95, 80, and 85 in Fig. 3. The susceptible population increase in starting time after-some time the population decreases rapidly with time. Next, Fig. 4 depicts the stability attainment for varying sizes of the acute infected class such as 20, 18, 16, and 14. Time to gain stability increases with the decrease in the population of the acute infected class. Figure 5. present the relationship between stability and varying extent of the chronic infected class for values such as 15, 12, 9, and 6. In starting the population decreases rapidly after 65 days population is constant with time. In Fig. 6 The recovered class is varied for values as, 4, 5, 3, and 2. The recovered population increase starting time after-some time the population decreases rapidly with time. Lastly, Fig. 7 shows the relationship between stability and vaccinated population for values such as 10, 8, 6, and 4. Vaccinated class increase in starting time after a short time the population decreases rapidly with time.

## Optimal control strategy

This section is dedicated to exploring the applicability of the infectious disease model with respect to the optimal control strategy. The employment of optimal control is initiated to intervene in the transmission of infectious disease in a particular community. Control strategies are formulated by employing sensitivity analysis and examining the dynamics of the proposed model. The maximum sensitivity index parameter is $(\gamma _1,\beta )$ whose value is between the interval (0.439, 1) increase in this parameter by 10 percent would increase the threshold quantity by (4.39, 1). Therefore, by using the control variable $u_1 (t)$,  $u_2(t)$, and $u_3(t)$, we must reduce these values to stop the sickness from spreading. $u_1(t)$ and $u_2(t)$ represent (awareness about medication, isolation, and ventilation). We employ the control of another variable $u_3(t)$ signify (the vaccination rates and quarantine level). Here, our aim is to reduce the infectious population by increasing the number of recovered people and decreasing the number of infected people and vaccinated people by incorporating the time-dependent control parameters, $u_1(t),\ \,u_2(t),\ \,\text {and}\ \,u_3(t)$ which are described as

- $u_1(t)$ is the control variable that characterizes the awareness about medication.
- $u_2(t)$ is the control variable that characterizes the isolation and ventilation.
- $u_3(t)$ is the control variable that characterizes the Vaccination rates and quarantine level.

The differential equation of the model (1), with the inclusion of the control variables, is, now given as

19 $$\begin{aligned} {\left\{ \begin{array}{ll} \begin{aligned}{}&{} \frac{d{\mathbb {S}}}{dt}=(1-u)\alpha -\beta ({\mathbb {I}}_1+\gamma {\mathbb {I}}_2){\mathbb {S}}(1-u_1)-d{\mathbb {S}}+\phi {\mathbb {R}}\\ {} &{}\,\,\,\,\,\,\,\,\,\,+\sigma {\mathbb {V}}+\rho _1\gamma _2{\mathbb {I}}_1+\rho _2\gamma _3{\mathbb {I}}_2,\\ &{} \frac{d{\mathbb {I}}_1}{dt}=\beta ({\mathbb {I}}_1+\gamma {\mathbb {I}}_2){\mathbb {S}}(1-u_1)-(\gamma _1+\gamma _2+d){\mathbb {I}}_1-{(u_2+u_3){\mathbb {I}}_1},\\ &{} \frac{d{\mathbb {I}}_2}{dt}=\gamma _1 {\mathbb {I}}_1-(d+d_1+\gamma _3){\mathbb {I}}_2-{(u_2+u_3){\mathbb {I}}_2},\\ &{} \frac{d{\mathbb {R}}}{dt}=(1-\rho _1) \gamma _2{\mathbb {I}}_1+(1-\rho _2) \gamma _3 {\mathbb {I}}_2-(\phi +d{+u_3) {\mathbb {R}}},\\ &{} \frac{d{\mathbb {V}}}{dt}=u\alpha -(\sigma +d) {\mathbb {V}}-{u_1}{\mathbb {V}}, \end{aligned} \end{array}\right. } \end{aligned}$$

along with the initial conditions are

$$\begin{aligned} {\mathbb {S}}(0) \ge 0,\,{\mathbb {I}}_1(0) \ge \,0, \,{\mathbb {I}}_2(0) \ge 0, \,{\mathbb {R}}(0) \ge 0,\, {\mathbb {V}}(0) \ge 0. \end{aligned}$$

The objective of this work is to show that time-dependent parameters minimize the cost of implementing those control measuring techniques^46^. In optimal control of infectious disease models, utilizing three classes of objective functions allows for a comprehensive assessment of intervention strategies. By considering objectives related to minimizing infections, controlling transmission rates, and maximizing the number of recovered people. Therefore, the objective function is specified as

20 $$\begin{aligned} J\big (u_1(t),u_2(t),u_3(t)\big ) = \int _{0}^{T}\bigg \{z_1 {\mathbb {I}}_1+ z_2 {\mathbb {I}}_2+z_3 {\mathbb {V}}+\frac{1}{2}\big (z_4 \,u_1^{2}(t)+z_5\, u_2^{2}(t)+z_6\, u_3^{2}(t)\big )\bigg \}\ \,dt, \end{aligned}$$

where, $z_1,\ \, z_2,\ \, z_3,\ \, z_4,\ \, z_5,\ \, \text {and}\ \, z_6,$ represents weight constant associated with each class. The weight constant $z_1,\ \, z_2,\ \, z_3,$ represents the cost of acute infected people, chronic infected people, and vaccinated people, while $z_4,\ \, z_5,\ \, z_6,$ represents the control variables. Further, $\bigg \{\frac{1}{2}z_4\ u_1^{2},\frac{1}{2}z_5 u_2^{2},\frac{1}{2} z_6u_3^{2}\bigg \}$ describes the awareness about medication, isolation and ventilation, vaccination rates and quarantine level. Our objective is to find the optimal control variables $u_1^{*},\ \, u_2^{*},\ \,u_3^{*}$ such that

21 $$\begin{aligned} J(u_1^{*}, u_2^{*},u_3^{*})=min\bigg \{J(u_1,u_2,u_3),u_1,u_2,u_3\epsilon U\bigg \}, \end{aligned}$$

dependent on control system (19), where we define the control set *U* as

22 $$\begin{aligned} U = \bigg \{(u_1,u_2,u_3)/u_i(t)\ \, \, \text {is lebesgue measurable on } [0,1],\, 0 \le \ \,u_i(t) \le 1,\, i = 1,2,3\bigg \}. \end{aligned}$$

### Existence of the optimal control system

The existence of optimal control is persuaded by considering initial time $t = 0$ for the fulfillment of each condition of the control system. To achieve the goal, the bounded Lebesgue measurable control^47^ by considering the initial conditions and ensuring an upper-bound solution for the system. The matter of optimal control is addressed by delving into the Lagrangian and Hamiltonian, whereas the following equation illustrates the optimal control problem within the Lagrangian framework as

$$\begin{aligned} {\left\{ \begin{array}{ll} L\big \{({\mathbb {S}},{\mathbb {I}}_1,{\mathbb {I}}_2,{\mathbb {R}},{\mathbb {V}},u_1,u_2,u_3)\big \} = z_1 {\mathbb {I}}_1 + z_2 {\mathbb {I}}_2\\+ z_3 {\mathbb {V}}+\frac{1}{2}\big (z_4\ \, u_1^{2}(t)+z_5\ \, u_2^{2}(t)+z_6\ \, u_3^{2}(t)\big ). \end{array}\right. } \end{aligned}$$

The minimal possible value of optimal control is achieved by providing the Hamiltonian function *H* in the subsequent form

$$\begin{aligned} H = L({\mathbb {S}},{\mathbb {I}}_1,{\mathbb {I}}_2,{\mathbb {R}},{\mathbb {V}},u_1,u_2,u_3) + \lambda _1\frac{d{\mathbb {S}}(t)}{dt} + \lambda _2\frac{d{\mathbb {I}}_1(t)}{dt} + \lambda _3\frac{d{\mathbb {I}}_2(t)}{dt} +\lambda _4\frac{d{\mathbb {V}}(t)}{dt}+\lambda _5\frac{d{\mathbb {R}}(t)}{dt}. \end{aligned}$$

Henceforth in this way the presence of optimal control, we consider the subsequent resultant. First we have to show that $(u_1^{*},u_2^{*},u_3^{*})$ these control variables are exist.

#### Theorem 5.1

There exists an optimal control $U^* = (u_1^{*},u_2^{*},u_3^{*})\ \,\epsilon U,$ to the control problem (19) and objective function (20).

#### Proof

In order to prove the existence of an optimal control, thru the result in^48^. Subsequently, the state variables and the control variables have all positive values. It is also detected that the control variable set *U* is convex and closed by the declaration. Furthermore, the control system is bounded which states the compactness of the considered problem. One may notice the convex nature of integrand involves in $\big [z_1 {\mathbb {I}}_1+ z_2 {\mathbb {I}}_2+z_3 {\mathbb {V}}+\frac{1}{2}(z_4\, u_1^{2}(t)+z_5 \,u_2^{2}(t)+z_6\, u_3^{2}(t))\big ]$ concerning control set *U* which guarantees the existence of the optimal control $(u_1^{*},u_2^{*},u_3^{*})$. $\square$

### Optimality condition

To define an optimal approach to the problem (19) and (20). First, we investigate the optimal control issues of Eqs. (19) and (20) using Lagrangian and Hamiltonian functions. The Lagrangian shows the optimal control problem is presented by the subsequent equation

$$\begin{aligned} {\left\{ \begin{array}{ll} L({\mathbb {I}}_1,{\mathbb {I}}_2,{\mathbb {V}},w_1,w_2,w_3) = \bigg \{z_1 {\mathbb {I}}_1 + z_2 {\mathbb {I}}_2\\ +z_3 {\mathbb {V}}+\frac{1}{2}\big (z_4\, u_1^{2}(t)+z_5\, u_2^{2}(t)+z_6\, u_3^{2}(t)\big )\bigg \}. \end{array}\right. } \end{aligned}$$

The associated Hamiltonian (*H*) is defined via the following notion $F = (F_1,F_2,F_3,F_4,F_5)$ and $\lambda =(\lambda _1,\lambda _2,\lambda _3,\lambda _4,\lambda _5)$. For the minimal value of the Lagrangian, we govern the Hamiltonian (*H*) for the optimal control problem by way of

$$\begin{aligned} H(x,u,\lambda ) = L(x,u)+\lambda F (x,u), \end{aligned}$$

where,

$$\begin{aligned} {\left\{ \begin{array}{ll} x = ({\mathbb {S}},{\mathbb {I}}_1,{\mathbb {I}}_2,{\mathbb {R}},{\mathbb {V}}),\,\,\,\, \lambda =(\lambda _1,\lambda _2,\lambda _3,\lambda _4,\lambda _5),\\ F(x,u) = F_1(x,u),F_2(x,u),F_3(x,u),F_4(x,u),F_5(x,u). \end{array}\right. } \end{aligned}$$

Further,

23 $$\begin{aligned} {\left\{ \begin{array}{ll} F_1(x,u)= (1-u)\alpha -\beta ({\mathbb {I}}_1+\gamma {\mathbb {I}}_2){\mathbb {S}}(1-u_1)-d{\mathbb {S}}+\phi {\mathbb {R}}+\sigma {\mathbb {V}}+\rho _1\gamma _2{\mathbb {I}}_1+\rho _2\gamma _3{\mathbb {I}}_2,\\ F_2(x,u) = \beta ({\mathbb {I}}_1+\gamma {\mathbb {I}}_2){\mathbb {S}}(1-u_1)-(\gamma _1+\gamma _2+d){\mathbb {I}}_1{-(u_2+u_3){\mathbb {I}}_1},\\ F_3(x,u) = \gamma _1 {\mathbb {I}}_1-(d+d_1+\gamma _3){\mathbb {I}}_2 {- (u_2+u_3{\mathbb {I}}_2,}\\ F_4(x,u) = (1-\rho _1)\gamma _2 {\mathbb {I}}_1 + (1-\rho _2)\gamma _3 {\mathbb {I}}_2 - (\phi +d+{u_3)}{\mathbb {R}},\\ F_5(x,u) = u\alpha - (\sigma +d){\mathbb {V}} {-u_1{\mathbb {V}}}.\\ \end{array}\right. } \end{aligned}$$

We revive the use of the Pontryagin maximum principle^49,50^ for finding the optimal solution to the proposed model (19). Further, the existence of the non-trivial vector functions such as $\lambda = (\lambda _1,\lambda _2,\lambda _3,\lambda _4,\lambda _5)$ is noticeable, especially when $(x^*,u^*)$ is considered as the optimal procedure for the launched control problem in regarding

$$\begin{aligned} \begin{aligned} \frac{dx}{dt} = \frac{\partial H(t, x, u, \lambda )}{\partial u},\\ 0=\frac{\partial H(t, x, u, \lambda )}{\partial u},\\ \lambda ^{'}(t) = -\frac{\partial H(t, x, u, \lambda )}{\partial x}.\\ \end{aligned} \end{aligned}$$

The Maximality condition

24 $$\begin{aligned} H(t,x^*(t),u^*(t),\lambda (t))\partial x= max_{u_1,u_2,u_3\epsilon [0,1]} H(x^*(t),u_1,u_2,u_3,\lambda (t)), \end{aligned}$$

describe the transversally condition as $\lambda (t_f)=0$.

#### Theorem 5.2

Given an optimal state $(u_1^*,u_2^*,u_3^*)$ variables and solution ${\mathbb {S}}^*,{\mathbb {I}}_1^*,{\mathbb {I}}_2^*,{\mathbb {R}}^*,{\mathbb {V}}^*$ of the equivalent state systems (19) and (20), there exist adjoint variables $\lambda (t)$ satisfying

25 $$\begin{aligned} {\left\{ \begin{array}{ll} { {\lambda }_1^{'} = \{(\lambda _1(1-u_1)\beta ({\mathbb {I}}_1+\gamma {\mathbb {I}}_2)-d)+\lambda _2\beta ({\mathbb {I}}_1+\gamma {\mathbb {I}}_2)(1-u_1)\},}&{}\\ { \lambda _2^{'} = \{z_1- (\lambda _1 (1-u_1)\beta {\mathbb {S}}+\rho _1\gamma _2)+(\lambda _2(\beta {\mathbb {S}}(1-u_1)-(\gamma _1+\gamma _2+d)-(u_2+u_3))+(\lambda _3\gamma _1)+(\lambda _4(1-\rho _1)\gamma _2)\},}&{}\\ { \lambda _3^{'} = -\big \{z_2-(\lambda _1(1-u_1)\beta \gamma {\mathbb {S}}+\rho _2\gamma _3)+\lambda _2(\gamma \beta {\mathbb {S}}(1-u_1))-\lambda _3[(d+d_1+\gamma _3)-(u_2+u_3)]+\lambda _4[(1-\rho _2)\gamma _3]\big \},}&{}\\ { \lambda _4^{'} = -\{\lambda _1\phi -\lambda _4(\phi +d+u_3\},}&{}\\ { \lambda _5^{'} = - \{ z_3+ \lambda _1\sigma -(\sigma +d+ u_1)\lambda _5\}.} \end{array}\right. } \end{aligned}$$

the transversality condition is defined as $\lambda _i(t) = 0 \, for \, i=1,2,3,4$.

Moreover, the control variables $u_1^*(t),\,u_2^*(t),\,u_3^*(t)$ are obtained as:

26 $$\begin{aligned} {\left\{ \begin{array}{ll} {u_1^{*}(t) = \max \bigg \{\min \bigg \{\frac{\beta ({\mathbb {I}}_1+\gamma {\mathbb {I}}_2){\mathbb {S}}(\lambda _1+\lambda _2)}{z_4},1\bigg \},0\bigg \},} &{}\\ {u_2^{*}(t) = \max \bigg \{\min \bigg \{\frac{{\mathbb {I}}_1\lambda _2+{\mathbb {I}}_2\lambda _3}{z_5},1\bigg \},0\bigg \},}&{}\\ {u_3^{*}(t) = \max \bigg \{\min \bigg \{ \frac{\lambda _2 {\mathbb {I}}_1+\lambda _3 {\mathbb {I}}_2+ \lambda _4(\phi +d)}{z_6},1\bigg \},0\bigg \}.} \end{array}\right. } \end{aligned}$$

#### Proof

**Figure 8:**
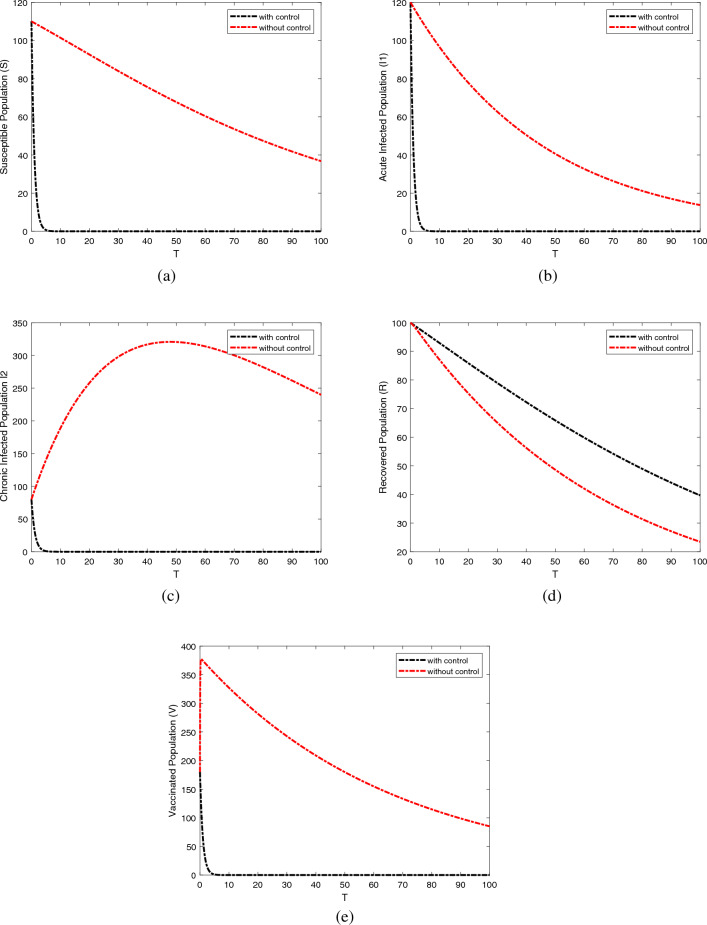
The graphical results display the dynamics behavior of the compartmental population are Susceptible, Acute, and Chronic Infected populations, Recovered and vaccinated with and without controls scenarios.

The Pontryagin Maximum Principle is directly applied to produce the adjoint system (25), while $\lambda (t) = 0$, directly results in the transversal condition. Regarding the class of optimal functions $u_1^{*}, u_2^{*}$ and $u_3^{*}$, we used $\frac{\partial H}{\partial u}$. In the section that follows, we numerically solve the optimality system. The control system defines the optimality system (19) with the adjoint system (25), boundary circumstances, as well as the best control strategies. Our control strategies, which are illustrated in Figures 8 and 10, to increase the susceptible and recovered population and reduce the infected and vaccinated population were supported by the simulation that was run. $\square$

### Numerical simulation and discussion

This section is dedicated to delineating the legitimacy of the proposed control scheme through the launch of numerical evaluation. The strategic environment is enriched by involving a diverse and wide range of parametric settings. The foundations of the numerical proceedings are instigated by considering the compartmental initial conditions are taken from^34^ for the devised model such as ${\mathbb {S}}(0) = 100,\ \,\, {\mathbb {I}}_1(0) = 20,\,\,\ {\mathbb {I}}_2(0) = 15,\ \, \, {\mathbb {V}}(0)=10$ and $R(0) = 5$. Moreover, the objective of consistency is maintained by taking into account the literature-oriented values of parameters associated with different compartments given in Table 3. We determine the ideal control system (19) to see the impact of medication, ventilation, isolation, and quarantine level. The resolve of the issue of optimization is persuaded through the employment of the Runge–Kutta fourth-order technique along with the application of the transversality criterion within the time interval of [0, 50]. Furthermore, the values of weight constant are assumed to be $z_1^{*}= 0.90030,\,\,\ z_2^{*} =0.43454,\,\,\ z_3^{*}=0.3550,\,\,\ z_4^{*}=0.5560,\,\,\ z_5^{*}=0.67676$ and $z_6^{*}=0.999$. Graphical representation and table of parameters used in Optimal control analysis are given below:

**Table 3:** Parameters and its values.

Parameters	Values	Source	Parameters	Values	Source
$\beta$	0.005	^34^	$\gamma$	0.2	Estimated
*u*	0.05	^35^	$\alpha$	0.01	^35^
$\rho _1$	0.78	^35^	$\rho _2$	0.93	^34^
*d*	0.02	^34^	$\sigma$	0.05	^35^
$d_1$	0.04	^34^	$\gamma _1$	0.065	^34^
$\gamma _2$	0.59	Estimated	$\gamma _3$	0.065	^34^
$\rho _3$	0.65	^34^	$\phi$	0.4	Estimated

**Figure 9:**
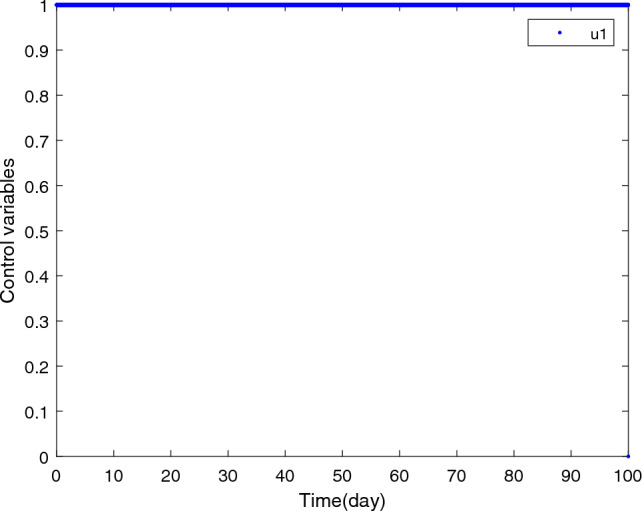
The graphical results display the dynamics behavior of control variable $u_1$.

**Figure 10:**
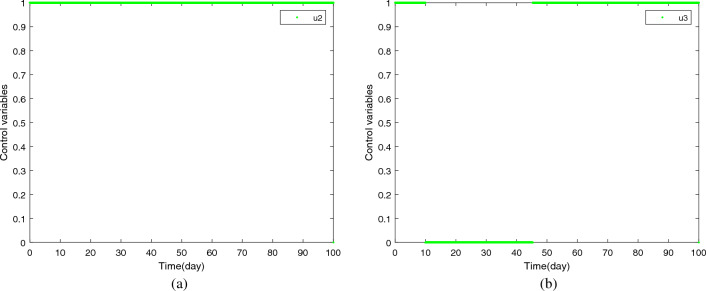
The graphical results display the dynamics behavior of control variables $u_2, u_3$.

Figures 8, 9, 10 depict the dynamics of comparative effectiveness of the proposed model, with respect to both scenarios that is with control and without control. This description is foreseen for all five compartments with respect to time in days. The gain of the optimal control strategy is noticeable for all compartments of the population. Figures 8 shows the dynamic behavior of Susceptible class ${\mathbb {S}}(t)$, Acute infected class ${\mathbb {I}}_1(t),$ Chronic infected class ${\mathbb {I}}_2(t)$, and Recovered class ${\mathbb {R}}(t)$ and the Vaccinated class ${\mathbb {V}}(t)$ with and without control interventions. Further, Fig. 8a represents the behavior of number of susceptible population decrease after using the optimal control strategy. Figure 8b Utilizing these control measures will reduce the number of acute infected populations after applying the control. Similarly, Fig. 8c the number of population of chronic infected populations decreased after applying the control. Figure 8d the recovered population rapidly increases and achieves convergence after applying optimal control. Figure 8e the number of the vaccinated population rapidly decreases after applying the control. The recovered population increase, reducing the infected and vaccinated population was evidently supported by the simulation that was run. Figure 10b,c,d are dedicated to highlighting the robustness of control variables with respect to time in days.

## Conclusion

This article proposes a novel mathematical model to encapsulate the viral transmission of infectious diseases. The optimal control strategy is launched while considering five related epidemiological compartments. The population under study is divided into five strata such as; Susceptible class ${\mathbb {S}}(t)$, Acute Infected class ${\mathbb {I}}_1(t)$, Chronic Infected class ${\mathbb {I}}_2(t)$, Recovered class ${\mathbb {R}}(t)$ and Vaccinated class ${\mathbb {V}}(t)$. The target of generality is maintained by employing a wide range of parametric settings involving, disease transmission rate from the susceptible class to the acute infected class, the untreated death rate of the susceptible class, the disease-related death rate of the acute infected class, a recovery rate of the chronic infected class. In the procession, rigorous persuasion into the local stability of the system, global stability of the system along with sensitivity analysis, positivity, and invariant region is considered. The implementations of the outcomes of the research are further supported and widened by the inclusion of more popular health interventions such as; quarantine, vaccination, medication, and mandatory use of masks. It is delineated that in the case of stability of the system, the susceptible class increase in starting point after some time population decrease rapidly with respect to time. Moreover, under the norm of stability, the interaction of underlying compartments indicates a higher likelihood of a decrease in the number of Acute-infected and Chronic-infected individuals. The exploration into the sensitivity parameter indicated an increase of 4.39 units in threshold quantity remained associated with a 10 percent increase in the sensitivity parameter.

For optimal control analysis, we focused on the introduction of parameters represented by $u_1, u_2$, and $u_3$, which are time-dependent control variables. We established an optimal control problem by defining an objective function with the goal of finding the optimal values for the afore mentioned control variables to minimize the overall cost. Applying Pontryagin’s principle, we derived the essential conditions for the optimal solution. The study explored the feasibility of three distinct optimal control approaches. In the first scenario, we determined optimal solutions by focusing solely on awareness about medication $u_1$ as a control variable. The second approach involved considering the effectiveness of isolation and quarantine $u_2$ as time-dependent controls. Lastly, the third approach involved vaccination rates $u_3$ as time-dependent controls. Graphical results clearly demonstrate the effectiveness of each strategy in reducing both acute and chronic infections, suggesting their potential implementation to mitigate the spread of infectious diseases in the population. The graphical results also revealed that the case where we considered all three control variables, simultaneously, is more effective in reducing the spread of infectious flow. The optimal control strategy advocated the efficacy of the employment of popular health interventions such as; quarantine, face masks, and hand washing, to restrict the transmission chain of the infectious disease. Additionally, our findings concluded that simulations with time-dependent controls are more cost-effective compared to those with time-independent controls.

In the future, it will be interesting to extend the above-developed model to explore the impact of the fractional order approach while maintaining the optimal control in place. It is anticipated that the ability of the fractional order scheme to use available information will be beneficial to further uncover the transmission of diseases in the population. Moreover, rigorous focus will remain on the exploration of the applicability of the proposed scheme on data sets involving varying geographical locations, socio-economic stratifications prevalent in the society along the degree of access to health facilities. Furthermore, it will be engaging to explore the efficacy of the devised model when dealing with multiple infectious flows at the same time. By figuring out how these strategies work together or against each other, we hope to create better plans to control these diseases around the world.

### Ethical approval

In this study, human data has not been used for modeling.

## Data Availability

The datasets generated during the current study are available from the corresponding author (Homan Emadifar) on reasonable request.
